# Left heart chambers reverse remodeling after combined CABG and mitral repair

**DOI:** 10.1186/1749-8090-8-S1-O215

**Published:** 2013-09-11

**Authors:** M Slavov, P Panayotov, D Panayotova, Y Peychev, V Kornovski

**Affiliations:** 1 Department of Cardiac Surgery, "St Marina" University Hospital, Varna, Bulgaria

## Background

Up to 30 % of all ischemic heart disease patients present with some degree of ischemic mitral regurgitation. It is proven that any grade adversely affects long-term outcomes. Surgical revascularization and restoration of the valvular function both trigger left atrial and ventricular reverse remodeling and improve the prognosis.

## Methods

To evaluate the actual reverse remodeling and its influence on the outcomes we perform follow-up on 71 patients subjected to combined surgical revascularization and mitral valve repair for mild to moderate ischemic mitral regurgitation. Mean follow-up was 28 (6 to 52) months. Left atrial and ventricular dimensions and volumes were evaluated preoperatively and at the follow-up.

## Results

The mean effective ejection fraction increased from 18 ± 6% to 39 ± 13% (p << 0.05) at the follow-up. Left ventricular end-systolic volume index decreased from 44 ± 24 ml/m2 to 39 ± 26 ml/m2 (p = 0.002). Significant left ventricular reverse remodeling (≥ 15% reduction of LVESVI) was observed in 55 % of all survivors. Recurrent IMR was detected in only 2.8% (2/71) of the evaluated patients. Left atrial volume index decreased from 43 ± 15 ml/m2 to 36 ± 14 ml/m2 (p = 0.000003) and left atrial reverse remodeling was significant for 57 % of all survivors.

## Conclusions

Despite the advanced process of left atrial and ventricular remodeling in the setting of chronic ischemic mitral regurgitation, left chambers can be effectively addressed by combined revascularization and mitral repair.

